# Wearables in der Rheumatologie

**DOI:** 10.1007/s00393-023-01377-8

**Published:** 2023-06-08

**Authors:** Tingting Xiong, Martin Krusche

**Affiliations:** Sektion für Rheumatologie und entzündliche Systemerkrankungen, III. Medizinische Klinik und Poliklinik, Universitätsklinikum Hamburg-Eppendorf, Martinistr. 52, 20246 Hamburg, Deutschland

**Keywords:** Smartwatch, Digitalisierung, Telemedizin, Monitorierung, Schrittzahl, Smartwatch, Digitisation, Telemedicine, Monitoring, Step number

## Abstract

Im Zuge der Digitalisierung der Medizin kommen Wearables eine zunehmende Bedeutung zu. Wearables (engl.: „wearable computing device“) sind kleine tragbare elektronische Geräte, über die der Anwender gesundheitsrelevante Daten wie Schrittzahl, Aktivitätsprofil, EKG, Herz- und Atemfrequenz oder Sauerstoffsättigung aufzeichnen kann. Erste Studien zum Einsatz von Wearables bei Patient:innen mit rheumatologischen Erkrankungen zeigen die Eröffnung von neuen Möglichkeiten zu Prävention, Krankheitsmonitorierung und Behandlung. Diese Arbeit soll eine Übersicht über die aktuelle Datenlage und den Einsatz der Wearables für das Fachgebiet der Rheumatologie liefern. Zusätzlich werden zukünftige potenzielle Anwendungsgebiete sowie Herausforderungen und Grenzen des Einsatzes von Wearables beleuchtet.

## Abstract (en)

As a result of digitalization in medicine wearable computing devices (wearables) are becoming increasingly more important. Wearables are small portable electronic devices with which the user can record data relevant to health, such as number of steps, activity profile, electrocardiogram (ECG), heart and breathing frequency or oxygen saturation. Initial studies on the use of wearables in patients with rheumatological diseases show the opening up of new possibilities for prevention, disease monitoring and treatment. This study provides the current data situation and the implementation of wearables in the discipline of rheumatology. Additionally, future potential fields of application as well as challenges and limits of the implementation of wearables are illustrated.

Der zunehmende Einzug der Digitalisierung in das Gesundheitswesen eröffnet neue Möglichkeiten in der Prävention, Diagnostik, Therapie sowie im Daten- und Informationstransfer in der Medizin. Neben der digitalen Patientenakte, Videosprechstunden oder Gesundheits-Apps kommt auch Wearables eine wachsende Bedeutung zu [1]. Wearables (engl.: „wearable computing device“) sind am Körper getragene Computer- oder Sensorsysteme, die gesundheitsrelevante Daten der tragenden Person und ihrer Umwelt aufzeichnen können. Sie ermöglichen eine Echtzeiterfassung von Daten, welche durch eine Übertragung mittels WLAN, Bluetooth oder Mobilfunknetz in einer Cloud gespeichert, weiterverarbeitet und über externe Endgeräte, wie z. B. Smartphones, eingesehen werden können. Der Markt für Wearables ist in den letzten Jahren stetig gewachsen: Wurden im Jahr 2014 weltweit noch 28,8 Mio. davon verkauft, waren es 2021 schon mehr als 533 Mio. [2]. Aktuell sind die meisten der Wearables zwar noch Lifestyleprodukte, jedoch nimmt ihre Bedeutung für medizinische Zwecke zunehmend zu. Einige Wearable-Hersteller konnten bereits für einzelne Gesundheitsfunktionen eine CE-Zertifizierung erhalten.

Ein großes Potenzial in der Medizin bieten Wearables im Hinblick auf eine mögliche Telemonitorierung. Durch ihren Einsatz ist eine engmaschige und präzise Erfassung von gesundheitsrelevanten Daten möglich, die zudem durch die überwiegend passive Erfassungsmethode zu einer einfacheren und besseren Akzeptanz beim Patienten führen kann. Hierdurch ist zukünftig neben der Monitorierung von Krankheitsschüben auch die Erfassung des Therapieerfolges denkbar, was u. a. auch interessant für die Durchführung klinischer Studien sein kann. Perspektivisch ist somit eine Entwicklung weg von der bisherigen ambulanten Quartalsmedizin hin zu einem lückenlosen Verständnis von Krankheitsverläufen denkbar.

Ziel dieses Artikels ist es, einen Überblick zur aktuellen Datenlage und zum Einsatz der Wearables für das Fachgebiet der Rheumatologie zu geben. Zusätzlich werden zukünftige potenzielle Anwendungsgebiete sowie Herausforderungen und Grenzen des Einsatzes von Wearables beleuchtet.

## Funktionen

**Figure 1:**
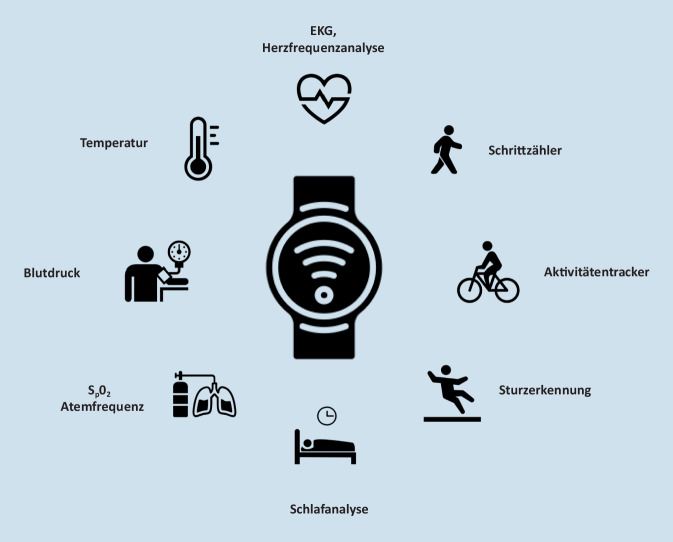

Zu den bekanntesten Wearables zählen Armbänder, Brustgurte sowie Smartwatches. Es existieren jedoch mittlerweile auch Wearables in Form von Datenbrillen, smarten Pflastern oder T‑Shirts. Ebenso vielfältig wie ihre Form sind die Funktionen, die Wearables ausführen können (Abb. 1): So können unter anderem die tägliche Schrittzahl, zurückgelegte Distanzen sowie Blutdruck und Herzfrequenz aufgezeichnet werden. Über EKG-Ableitungen sowie photoplethysmographische Messungen, welche die Pulsfrequenzvariabilität erfassen, können zudem Herzrhythmusstörungen wie Vorhofflimmern detektiert werden. Mittels integrierten Pulsoxymeters kann außerdem kontinuierlich die periphere Sauerstoffsättigung gemessen werden. Durch die kombinierte Auswertung der erfassten Daten, teils unterstützt durch KI-Algorithmen, können individuelle Aktivitätsprofile inklusive Sturzdetektion bis hin zur Schlafanalyse erstellt werden. Eine Übersicht über die Gesundheitsfunktionen aktueller Smartwatches unterschiedlicher Hersteller ist in Tab. 1 aufgeführt.

**Table 1:** 

Hersteller	Gesundheitsfunktionen	Preis
Fitbit Sense 2 (Fitbit, San Francisco, Kalifornien, USA)	Schrittzähler Aktivitätentracker EKG^a^ Temperaturmessung Schlafqualität Sauerstoffsättigung, Atemfrequenz	Ab 299 €
Samsung Galaxy Watch 4 (Samsung, Seoul, Südkorea)	Schrittzähler Aktivitätentracker EKG-Messung^a^ Blutdruckmessung^a^ Sauerstoffsättigung, Atemfrequenz Schlafanalyse Körperzusammensetzungsanalyse	Ab 349 €
Withings ScanWatch (Withings, Issy-les-Moulineaux, Frankreich)	Schrittzähler Aktivitätentracker Klinisch validiertes EKG^a^ Proaktiver Herz-Scan^a^ Sauerstoffsättigung^a^, Atemfrequenz, Scan auf Atemstörungen Schlafqualität	Ab 199 €
Garmin Venu 2 (Garmin, Olathe, Kansas, USA)	Schrittzähler Aktivitätentracker Herzfrequenzmessung Sauerstoffsättigung, Atemfrequenz Schlafqualität	Ab 299 €
Apple Watch Series 8 (Cupertino, Kalifornien, USA)	Schrittzähler Aktivitätentracker EKG^a^ Proaktiver Herz-Scan^a^ Sturzerkennung Temperaturmessung, Zykluserkennung Schlafanalyse Sauerstoffsättigung, Atemfrequenz	Ab 499 €

^a^CE-zertifizierte Gesundheitsfunktionen

## Einsatz in der Rheumatologie

Der Einsatz von Wearables in der Rheumatologie sowie die wissenschaftliche Auswertung entsprechender Daten befindet sich derzeit noch in den Anfängen [3]. Einige Arbeiten zeigen jedoch bereits einen potenziellen Mehrwert von Wearables für die Krankheitsmonitorierung und Behandlung von Patient:innen mit rheumatologischen Erkrankungen. Eine Übersicht ist in Tab. 2 dargestellt.

**Table 2:** 

Studie	Art der Studie	Erkrankung	Intervention mittels Wearable	Outcome
Ocagli (2022) [15]	Metaanalyse	7488 Patient:innen mit inflammatorischen und nichtinflammatorischen Arthropathien sowie rheumatologischen Systemerkrankungen	Erfassung Schrittzahl und MVPA	Nichterreichen des empfohlenen WHO-Tagesziels von 10.000 Schritten/Tag, jedoch der MVPA von 22 min/Woche in allen Subgruppen
Davergne (2019) [22]	Metaanalyse	1588 Patient:innen mit entzündlich rheumatischen Gelenkerkrankungen und muskuloskeletalen Erkrankungen	Erfassung Schrittzahl und MVPA	Steigerung der täglichen Schrittzahl um 1520 sowie Dauer MVPA um 16 min
Li (2020) [24]	RCT	118 Patient:innen mit RA und SLE (59 vs. 59)	Erfassung Schrittzahl und MVPA; Schulung und 4 Follow-up-Anrufe durch Physiotherapeut:in über 27 Wochen	Verbesserung MVPA um 14,3 min/Tag und Schmerzwahrnehmung bei Patient:innen mit RA, nicht bei SLE
Wang (2022) [26]	RCT	54 Patient:innen mit AS (26 vs. 28)	Wearable-unterstützte Durchführung von aeroben Trainingseinheiten über 16 Wochen	Verbesserung ASADS, PGA, PhGA, BASDAI, BASFI in Interventionsgruppe
Jacquemin (2017) [27]	Prospektive Beobachtungsstudie	79 Patient:innen mit axSpA und 91 mit RA	Erfassung Schrittzahl über 3 Monate Erfassung Krankheitsschübe mittels wöchentlicher Fragebögen	Anhaltende Schübe > 3 Tage assoziiert mit moderater Abnahme der Schrittzahl/Tag um 836 bis 1462
Gossec (2019) [28]	Prospektive Beobachtungsstudie	155 Patient:innen mit RA und axSpA	Erfassung Aktivitätsmuster über 3 Monate und Anwendung von maschinellen Lerntechniken	Anhand des Aktivitätsmusters konnten Krankheitsschübe mit einer Sensitivität von 95,7 % und einer Spezifität von 96,7 % detektiert werden
Elmagboul (2020) [29]	Prospektive Beobachtungsstudie	33 Patient:innen mit Gicht	Erfassung Schrittzahl und Schlafqualität	Innerhalb des Studienzeitraumes von 204 (38 %) Personenwochen mit Gichtanfall sowie 340 (62 %) Personenwochen ohne Gichtanfall Abnahme der Schrittzahl/Tag um 841 bei Patient:innen mit Gichtanfall, kein Unterschied der Schlafqualität
Oldroyd (2019) [30]	Narratives Review	181 Patient:innen mit inflammatorischen Myopathien	Erfassung des Aktivitätsprofils	Assoziation zwischen Aktivitätsprofil sowie Krankheitsdauer, aktueller Glukokortikoidtherapie und CK-Level, kein Zusammenhang zur Muskelkraft
Saygin (2021) [31]	Prospektive Beobachtungsstudie	24 Patient:innen mit inflammatorischen Myopathien	Erfassung Schrittzahl und maximal 1‑min-Schrittfrequenz	Schrittzahl/Tag und maximal 1‑min-Schrittfrequenz korreliert mit PGA, PhGA, HAQ-Behinderungsindex, SF-36 PF10 und Funktionstests (6-min-Gehtest, Timed-up-and-go-Test, „six-min walk“, Timed up-and-go, Sit-to-stand-Test)

*MVPA* „moderate to vigorous physical activity“, *RCT* randomisiert kontrollierte Studie, *RA* rheumatoide Arthritis, *SLE* systemischer Lupus erythematodes, *AS* ankylosierende Spondyloarthritis,* axSpA* axiale Spondyloarthritis, *ASDAS* Ankylosing Spondylitis Disease Acitivity Score, *PGA* „patient global assessment“, *PhGA* „physician global assessment“, *BASDAI* Bath Ankylosing Spondylitis Disease Activity Index, *BASFI* Bath Ankylosing Spondylitis Functional Index, *CK* Creatinkinase

## Physische Aktivität

Die regelmäßige körperliche Bewegung spielt eine wichtige Rolle in der Aufrechterhaltung körperlicher Gesundheit sowie geistigen Wohlbefindens. Zahlreiche Daten belegen eine Reduktion der Gesamtmortalität [4], Abnahme der Inzidenz von Erkrankungen wie Typ II Diabetes mellitus, Adipositas, Krebs, arterielle Hypertonie [5] sowie auch positive Einflussnahme auf die psychische Gesundheit [6] durch regelmäßige körperliche Aktivitäten. Metaanalysen zeigen, dass Patient:innen mit entzündlich rheumatischen Gelenkerkrankungen von körperlicher Aktivität insbesondere in Bezug auf die kardiovaskuläre Fitness und Muskelkraft profitieren [7]. Auch zeigte sich in einer Studie an Patient:innen mit rheumatoider Arthritis (RA), dass die körperliche Aktivität neben einer höheren Funktionskapazität (gemessen anhand des Funktionsfragebogens Hannover [FFbH]) auch mit weniger Fatiguesymptomatik assoziiert ist [8]. Die Bedeutung von regelmäßiger körperlicher Betätigung bei entzündlichen Arthritiden sowie Arthrose spiegelt sich auch in den Therapieempfehlungen der European League Against Rheumatism (EULAR) von 2018 wider [9]. Diese sind unter anderem angelehnt an die Empfehlungen zur sportlichen Aktivität der Weltgesundheitsorganisation (WHO) [10], welche eine mäßige körperliche Aktivität von mindestens 150 min pro Woche oder intensive körperliche Aktivität von 75 min pro Woche empfiehlt. Laut aktuellen WHO-Richtlinien werden 10.000 Schritte pro Tag für gesunde Individuen empfohlen [11, 12].

Bei Patient:innen mit rheumatischen Erkrankungen des Bewegungsapparates kann die körperliche Aktivität aufgrund von akuter Inflammation, chronischem Erkrankungsverlauf mit Funktionseinschränkungen sowie damit einhergehenden psychischen Belastungen reduziert sein [13, 14].

Eine Metaanalyse von Ocagli et al. wertete untersuchte Wearabledaten von insgesamt 7488 Patient:innen mit inflammatorischen und nichtinflammatorischen Arthropathien sowie rheumatologischen Systemerkrankungen aus. Hierbei zeigte sich, dass alle untersuchten Gruppen deutlich unter den von der WHO empfohlenen 10.000 Schritten pro Tag blieben (autoimmune Systemerkrankungen durchschnittlich 5130 Schritte pro Tag, Fibromyalgie 6045, Osteoarthritis 5460, inflammatorische Arthropathien 6360), während hingegen die empfohlene Dauer von etwa 22 min pro Tag an körperlicher Aktivität mit moderater bis hoher Intensität von allen Subgruppen erreicht wurden (autoimmune Systemerkrankungen durchschnittlich 34,75 min pro Tag, Fibromyalgie 67,5, Osteoarthritis 29,8, inflammatorische Arthropathien 45,7) [15].

## Steigerung der physischen Aktivität durch Wearables

Neben eingeschränktem physiotherapeutischem Angebot [16] können auch mangelnde Gesundheitskompetenz [17] sowie fehlende Motivation [18] eine Steigerung der körperlichen Betätigung bei rheumatologischen Patient:innen erschweren. Hier könnte sich der ergänzende Einsatz von Wearables als sinnvoll erweisen, da sie aufgrund des Designs, der Nutzerfreundlichkeit [19] sowie kontinuierlicher passiver Datenerfassung mit Möglichkeit zur individuellen Zielsetzung mit Erinnerungsnachrichten [20] die Hürden zur körperlichen Betätigung im Alltag verringern und die Motivation steigern können.

Eine kürzlich publizierte systematische Übersichtsarbeit [21] mit Datenanalyse von über 160.000 Studienteilnehmern zeigte, dass Probanden, die ein Wearable trugen im Schnitt 1800 Schritte pro Tag, 40 min Gehzeit sowie 6 min körperliche Aktivität von moderat bis hoher Intensität mehr durchführten als Personen, die einen vergleichbaren Lebensstil führten. Allerdings war der Effekt auf andere physiologische (Blutdruck, Cholesterinspiegel, HbA_1c_-Wert) sowie psychosoziale Endpunkte (Lebensqualität, Schmerz) laut der Analyse gering und häufig nicht signifikant.

Die Daten einer Metaanalyse von 1588 Patient:innen mit entzündlich rheumatischen Gelenkerkrankungen und muskuloskeletalen Erkrankungen belegen, dass der Einsatz von Aktivitätstrackern über einen Zeitraum von 10 Wochen bei einer insgesamt hohen Adhärenz (92,7 % Tragezeit) zu einer signifikanten Steigerung der durchschnittlichen täglichen Schrittzahl um 1520 sowie gesteigerten Dauer von 16 min körperlicher Aktivität von moderater bis hoher Intensität führte [22]. Eine 2022 veröffentliche Studie zeigte, dass eine Steigerung der körperlichen Aktivität mit moderater bis hoher Intensität um 10 min pro Tag mit einer Reduktion der Todesfälle pro Jahr von 6,9 % in der US-amerikanischen Bevölkerung assoziiert war [23]. Auch wenn sich innerhalb der kurzen Beobachtungszeit durch die mäßige Bewegungssteigerung kein direkter Effekt auf die Endpunkte Invalidität, funktionale Tests, Lebensqualität sowie Fatigue zeigte, zeigt diese Analyse dennoch, dass die zusätzliche Anwendung von Wearables auch bei Patient:innen mit rheumatologischen Grunderkrankungen einen potenziellen Nutzen in der Steigerung der physischen Aktivität haben kann. Dies bestätigte auch eine randomisiert kontrollierte Studie an 118 Patient:innen mit RA sowie systemischem Lupus erythematodes (SLE), deren körperliche Aktivität mittels Wearable monitoriert wurde und die zusätzlich telefonische Follow-up-Anrufe von Physiotherapeuten erhielten. Nach einer Beobachtungszeit von 27 Wochen zeigten sich eine gestiegene körperliche Aktivität sowie reduzierte Schmerzen in der Patientengruppe mit RA [24].

Neben der Steigerung der physischen Aktivität können Wearables auch einen Beitrag in der Monitorierung der Intensität derselbigen leisten. Zwar ist eine Steigerung der physischen Aktivität mit positiven gesundheitlichen Aspekten assoziiert, doch kann sich eine unkontrollierte zu hohe Trainingsintensität auch negativ auswirken. So haben beispielsweise Patient:innen mit axialer Spondyloarthritis (axSpA) durch Enthesitiden ein erhöhtes Risiko für Verletzungen im Rahmen starker Gelenkbelastungen oder raschem Wechsel von Inaktivität zu Aktivität [25]. Eine randomisierte Studie in dieser Patientengruppe zeigte, dass die Wearable-unterstützte Durchführung von aeroben Trainingseinheiten (64–76 % der maximalen Herzfrequenz) in der Häuslichkeit über einen Zeitraum von 16 Wochen zu einer signifikanten Besserung des Ankylosing Spondylitis Disease Activity Score (ASDAS) sowie Bath Ankylosing Spondylitis Disease Activity Index (BASDAI) und Bath Ankylosing Spondylitis Functional Index (BASFI) im Vergleich zu Patient:innen ohne kontrollierte Trainingseinheiten führte [26].

## Krankheitsmonitorierung

Neben dem Einsatz zur Steigerung der physischen Aktivität belegen erste Studiendaten, dass Wearables zukünftig auch in der Überwachung des individuellen Krankheitsverlaufs eine wichtige Funktion einnehmen können.

In einer prospektiven Beobachtungsstudie wurde die körperliche Aktivität von 79 Patient:innen mit axSpA sowie 91 Patient:innen mit RA kontinuierlich über 3 Monate mit einem Aktivitätstracker erfasst, und subjektive Krankheitsschübe wurden durch Fragebögen wöchentlich ermittelt. Die Autor:innen konnten zeigen, dass anhaltende Schübe > 3 Tage mit einer moderaten Abnahme der körperlichen Aktivität (Abnahme der Schrittzahl/Tag um 836 bis 1462) assoziiert waren [27]. Eine französische prospektive Multicenterstudie führte diesen Ansatz fort und wendete maschinelle Lerntechniken auf die gesammelten klinischen Daten von insgesamt 155 Patient:innen mit RA oder axialer Spondyloarthritis an, die ebenfalls über die 3‑monatige Studiendauer mit einem Aktivitätstracker ausgestattet waren [28]. Das maschinelle Lernprogramm konnte zuverlässig das Auftreten als auch die Abwesenheit von Krankheitsschüben mit einer Sensitivität von 95,7 % und einer Spezifität von 96,7 % detektieren.

Auch in einer Studie mit 33 Patient:innen mit vordiagnostizierter Gicht konnte innerhalb des Studienzeitraumes von 204 (38 %) Personenwochen mit Gichtanfall sowie 340 (62 %) Personenwochen ohne Gichtanfall eine signifikante Abnahme der durchschnittlichen täglichen Schrittzahl von 841 bei Patient:innen mit Gichtanfall festgestellt werden. Ein Einfluss von Gichtanfällen auf die Schlafdauer konnte hingegen nicht beobachtet werden [29]. Ebenso konnte ein narratives Review über 181 Patient:innen mit inflammatorischen Myopathien eine Assoziation zwischen dem Bewegungsprofil sowie Krankheitsdauer, aktueller Glukokortikoidtherapie und CK-Level feststellen. Ein Zusammenhang zwischen der Muskelkraft und dem Aktivitätsprofil konnte jedoch nicht nachgewiesen werden [30]. Eine prospektive Beobachtungsstudie mit 24 Patient:innen mit inflammatorischen Myopathien konnte eine Korrelation der täglichen Schrittzahl sowie maximalen 1‑min-Schrittfrequenz mit der Einschätzung der Krankheitsaktivität durch Arzt und Patient, dem HAQ-Behinderungsindex, SF-36 PF10 sowie verschiedenen Funktionstests (6-min-Gehtest, Timed up-and-go, Sit-to-Stand-Test) zeigen [31].

## Weitere potenzielle Anwendungsgebiete in der Rheumatologie

Bislang beschränkt sich die wissenschaftliche Auswertung von mittels Wearables erfassten Daten bei rheumatologischen Patient:innen weitestgehend auf die Erfassung von Schrittzahl sowie Aktivitätsmuster. Aufgrund der zunehmenden Messgenauigkeit sowie Datenanalyse mithilfe von künstlicher Intelligenz (KI) sind zukünftig darüber hinaus auch weitere Anwendungsgebiete in der Rheumatologie denkbar.

## Herzrhythmusstörungen

Zahlreiche Studien belegen, dass Patient:innen mit entzündlich rheumatischen Erkrankungen ein erhöhtes Risiko für Herzrhythmusstörungen aufweisen. So sind Patienten mit Kollagenosen wie systemischer Sklerose, Myositiden oder SLE im Rahmen einer kardialen Beteiligung verstärkt gefährdet [32–34]. Auch Patient:innen mit RA zeigen ein erhöhtes Risiko für Vorhofflimmern [35]. Zusätzlich können auch medikamentöse Therapien, wie z. B. Iloprost, zu einem erhöhten Arrhythmierisiko führen [36]. Da Herzrhythmusstörungen häufig asymptomatisch verlaufen können, ist eine frühe und korrekte Aufzeichnung wichtig, um rechtzeitig therapeutische Maßnahmen einleiten zu können und Folgeschäden wie Schlaganfälle zu vermeiden. Wearables in Form von Uhren oder Armbändern eröffnen neue Möglichkeit zur Arrhythmiedetektion. Im Wesentlichen kommen hierbei 2 Technologien zum Einsatz: Die Photoplethysmographie und die 1‑Kanal-Elektrokardiographie. In der Kardiologie hat die Wearable-basierte Detektion von Arrhythmien bereits früh den Eingang in wissenschaftliche Studien gefunden. Neben der Bestimmung der Herzfrequenz können mit einer mäßigen Erkennungsrate Asystolien, Bradykardien, Tachykardien, ventrikuläre Tachykardien und Kammerflimmern detektiert werden [37], wobei die belastbarsten Studiendaten zur Detektion des Vorhofflimmerns vorliegen. Eine der bisher bekanntesten Studien hierzu ist die Apple Heart Study [38]. In der über 400.000 Studienteilnehmer umfassenden Studie konnte nachgewiesen werden, dass bei Patient:innen mit Vorhofflimmern und mit Sinusrhythmus eine hohe Übereinstimmung des mittels der Apple Watch erfassten EKG mit einem konventionellen 12-Kanal-EKG bestand. Des Weiteren konnte in einer randomisiert kontrollierten Studie bei Patient:innen über 65 Jahren mit erhöhtem Schlaganfallrisiko belegt werden, dass ein 2‑mal wöchentliches Wearable-EKG der Routinediagnostik durch den Hausarzt in der Detektion eines Vorhofflimmerns überlegen war [39]. Zwar konnte ein weiterer Nutzen über die Diagnose hinaus bislang noch nicht gezeigt werden, jedoch betont die Deutsche Gesellschaft für Kardiologie in ihrem Positionspapier: „Wearables sind potenziell in der Lage die diagnostische Lücke zu schließen, die das konventionelle EKG-basierte Screening hinterlässt.“ [40]

## Sauerstoffsättigung

Neben kardialen Ereignissen kann die Prognose bei Patient:innen mit entzündlich rheumatischen Erkrankungen auch durch pulmonale Organbeteiligung wie interstitielle Lungenerkrankungen oder alveoläre Hämorrhagien erheblich negativ beeinflusst werden [41].

Studien konnten zeigen, dass eine hohe Korrelation (r = 0,81–0,89) von mittels Wearable erfassten Sauerstoffsättigungswerten im Vergleich zu konventionellen Sättigungsmessgeräten bei Patient:innen mit Lungenerkrankungen besteht [42, 43]. Eine Untersuchung an 83 Patient:innen mit Systemsklerose assoziierter interstitieller Lungenerkrankungen zeigte eine hohe Korrelation der peripher gemessenen Sauerstoffsättigung mit einer arteriellen Sauerstoffsättigungsmessung während körperlicher Trainingseinheiten, wobei eine Sauerstoffsättigung von unter 89 % oder Abfall über 4 Punkte mit einer erhöhten Mortalität assoziiert war [44]. Der Einsatz von Wearables in der Häuslichkeit (ambulantes Monitoring) könnte sich somit zur frühzeitigen Detektion von Sättigungsabfällen sowie erhöhten Atemfrequenzen beispielsweise im Rahmen von fortschreitender Lungenfibrose oder akuten pulmonalen Infekten sowie kardialer Dekompensation als sinnvoll erweisen.

## Schlafqualität

Schlafstörungen sind ein häufiges Problem bei Patient:innen mit rheumatologischen Erkrankungen und können zu einem chronischen Fatiguesyndrom führen [45]. Studiendaten zeigen, dass beispielweise über die Hälfte der Patient:innen mit Spondyloarthritiden unter Schlafstörungen leidet [46]. Neben einer schlechten Schlafqualität gehören Einschlaf- sowie Durchschlafstörungen und das obstruktive Schlafapnoesyndrom zu den häufigsten Schlafstörungen [47].

Der derzeitige Goldstandard zur Evaluation der Schlafqualität stellt die Polysomnographie dar, welche Aussagen über die Schlafarchitektur sowie schlafbezogene Atmungsmuster gibt [48]. Die Durchführung einer Polysomnographie erfolgt üblicherweise im Schlaflabor und ist mit höheren Kosten verbunden. Die Durchführung selbst kann zudem im Schlaf als störend empfunden werden und zeichnet nur einen limitierten Zeitraum auf, welcher je nach Krankheitsstadium nicht zwingend repräsentativ ist [48, 49]. Auch mittlerweile verfügbare mobile Schlaflabore für die häusliche Anwendung können die genannten Hindernisse nicht vollständig umgehen.

Daher bedarf es praktikablerer Lösungen, welche wenig invasiv sind und im Alltag auch über einen längeren Zeitraum umgesetzt werden können. Die bislang am weitesten verbreitete Methode, Schlafqualität zu erfassen, ist die Patientenbefragung mithilfe von Fragebögen, welche zwar kostengünstig umzusetzen ist, jedoch sowohl einem Reporting-Bias unterliegt [50] als auch durch Erinnerungsschwierigkeiten bei länger zurückliegenden Zeiträumen beeinflusst wird [51]. Zwar ist die Schlafaufzeichnung mittels Wearables nicht im vollen Umfang mit einer Polysomnographie im Schlaflabor zu vergleichen und auch die Genauigkeit der Auswertung bislang noch nicht ausreichend für medizinische Beurteilungen, so können doch mittlerweile bereits zahlreiche Informationen wie Tiefschlaf‑, Leichtschlaf‑, REM- sowie Wachphasen erfasst werden [52]. Auch können schlafbezogene Atmungsstörungen wie Schlafapnoephasen detektiert werden [53].

In prospektiven Studien bei Patient:innen mit RA sowie Arthrose erwiesen sich Wearables bereits als gute Möglichkeit, die Auswirkung von Therapieinterventionen auf die körperliche Aktivität sowie auch Schlafqualität objektiv zu erfassen [54, 55].

## Herausforderungen

Das große Potenzial medizinischer Wearables bringt auch einige Herausforderungen mit sich.

Durch die passive und kontinuierliche Erfassung zahlreicher Patientendaten im Alltag entsteht in der Gesamtsumme eine wahre „Datenflut“. Nicht alle Daten, die erfasst werden, haben letzten Endes eine klinische Relevanz und müssen über einen längeren Zeitraum gespeichert werden. Insbesondere vor dem Hintergrund einer zukünftig denkbaren telemedizinischen Übermittlung von durch Wearables generierten Daten an das Gesundheitssystem ist eine sorgfältige Vorselektion gesundheitsrelevanter Daten notwendig. Auch wird eine nutzerorientierte Aufklärung oder Schulung notwendig sein, um Überforderung mit einer zu großen Datenmenge oder Verunsicherung hinsichtlich der Dateninterpretation zu vermeiden [56].

Zwar zeigen die oben genannten Studien bereits ein vielversprechendes Potenzial von Wearables in der Krankheitsmonitorierung rheumatologischer Patient:innen, jedoch sind die bisher erfassbaren Daten ohne Zweifel leicht durch weitere Faktoren beeinflussbar. So könnten beispielweise Krankheitsschübe durch Komorbiditäten wie Depression und Traumata imitiert werden. Eine Weiterentwicklung hinsichtlich der Dateninterpretation ist somit zwingend erforderlich, auch um unnötige Vorstellungen im Gesundheitssystem zu minimieren.

Wearables mit Gesundheitsfunktionen, die über eine Schrittzählerfunktion hinausgehen, sind zudem derzeit für den Endnutzer noch verhältnismäßig kostenintensiv und werden bislang nicht regulär von der Krankenkasse erstattet. Sollte es zukünftig dazu kommen, dass Wearables in der Medizin eine relevante Rolle einnehmen, besteht das Risiko, dass es zur Benachteiligung bestimmter Patientengruppen aufgrund von sozioökonomischen Faktoren kommen kann. Auch kann eine Benachteiligung von Patientengruppen aufgrund unzureichender Digitalkompetenz entstehen.

Eine weitere übergeordnete Herausforderung im Rahmen der Digitalisierungsbewegung stellt die Datensicherheit dar, denn die Nutzung von Wearables birgt Risiken für das Recht auf informationelle Selbstbestimmung. Schrittdaten wirken auf den ersten Blick nicht sensibel, können jedoch, insbesondere wenn mehrdimensionale Daten wie Herzfrequenz, Schlaf- oder Standortdaten hinzukommen, zu sehr individuellen Profilen zusammengestellt werden. Diese könnten wiederum für Werbetreibende, Krankenkassen oder aber auch den Arbeitgeber von potenziellem Interesse sein. Die Speicherung sowie Übertragung von sensiblen personenbezogenen Gesundheitsdaten bedarf somit höchster Anforderungen an die Datensicherheit.

## Fazit

Wearables, die bislang überwiegend in der Freizeit als Lifestyle Gadgets ihre Anwendung gefunden haben, rücken zunehmend in den Fokus der Medizin. Durch die kontinuierliche und überwiegend passive Erfassung gesundheitsrelevanter Daten ermöglichen sie eine Echtzeitüberwachung von Erkrankten sowie das Sammeln wichtiger Patient:innendaten, die mithilfe von KI-gestützten Funktionen weiterverarbeitet werden können. Erste Studien zeigen bereits die Möglichkeit zum Krankheitsmonitoring bei Patient:innen mit rheumatologischen Erkrankungen. Wearables können bislang Arztbesuche, Diagnoseverfahren und Therapien zwar nicht ersetzen, können sie jedoch in Zukunft in einer zunehmend vernetzten medizinischen Versorgung mit elektronischer Gesundheitsakte und Telemedizinangeboten sinnvoll ergänzen und bergen das Potenzial, die personalisierte Medizin zu revolutionieren. Für die medizinische Forschung stellen von Wearables generierte Daten bislang eine neue Ressource dar. Zukünftig könnten größere Datenmengen von Wearables-Nutzern dabei helfen, rheumatologische Erkrankungen besser zu verstehen, die Früherkennung von Krankheiten zu fördern und Therapieformen zu optimieren.

Auch wenn man heute noch keine pauschale Empfehlung für den flächendeckenden Nutzen von Wearables in der Rheumatologie aussprechen kann, so legen Metaanalysedaten durchaus einen Mehrwert zur Steigerung von physischer Aktivität von Patient:innen nahe. Darüber hinaus kann die (Selbst‑)Monitorierung von gesundheitsbezogenen Daten zum Empowerment von Betroffenen beitragen, sodass nach Meinung der Autoren auch in unserem Fachgebiet der Einsatz von Wearables auf Wunsch individuell mit den Patient:innen besprochen werden sollte.
